# Comparative Evaluation of Postoperative Pain Following Nonsurgical Root Canal Treatment Using Calcium Hydroxide, Bio-C Temp, and Odontopaste as Intracanal Medicaments: A Prospective Clinical Study

**DOI:** 10.7759/cureus.115284

**Published:** 2026-08-27

**Authors:** Sameer Chauhan, Saloni Jain, Saksham Narainia, Nehal S Sonawane, Anirban Das, Neelam Kukreja

**Affiliations:** 1 Department of Prosthodontics, K. M. Shah Dental College and Hospital, Sumandeep Vidyapeeth (Deemed to be University), Vadodara, IND; 2 Department of Conservative Dentistry and Endodontics, Bharati Vidyapeeth (Deemed to be University) Dental College and Hospital, Sangli, IND; 3 Department of Conservative Dentistry and Endodontics, Indira Gandhi Government Dental College and Hospital, Jammu, IND; 4 Department of Conservative Dentistry and Endodontics, Yogita Dental College and Hospital, Khed, IND; 5 Department of Pedodontics and Preventive Dentistry, Kusum Devi Sunderlal Dugar Jain Dental College and Hospital, Kolkata, IND; 6 Department of Conservative Dentistry and Endodontics, Lenora Institute of Dental Sciences, Rajahmundry, IND

**Keywords:** bioceramic materials, calcium hydroxide, intracanal medicaments, postoperative pain, root canal therapy

## Abstract

Background: Postoperative pain remains one of the most common complications following nonsurgical root canal treatment, and the choice of intracanal medication may influence the severity and duration of postoperative discomfort. This study compared the effectiveness of calcium hydroxide, Bio-C Temp (Angelus Indústria de Produtos Odontológicos S.A., Londrina, Brazil), and Odontopaste (Australian Dental Manufacturing Pty. Ltd., Brisbane, QLD, Australia) in controlling postoperative pain.

Methodology: This prospective clinical study was conducted on 180 participants who required nonsurgical root canal treatment and were equally divided into three groups (60, 33.3%, each): calcium hydroxide, Bio-C Temp, and Odontopaste. Root canal treatment was performed using a standardized protocol. Postoperative pain was assessed using a 100-mm visual analog scale (VAS) at 24 hours, 48 hours, 72 hours, and seven days. The secondary outcomes included analgesic consumption, time to first analgesic intake, interappointment flare-up, postoperative swelling, and percussion tenderness. Data were analyzed using one-way analysis of variance (ANOVA), Kruskal-Wallis test, chi-square test, linear mixed-effects model, Kaplan-Meier survival analysis, and multivariable linear regression, with statistical significance set at *P* < 0.05.

Results: Baseline demographic and clinical characteristics were comparable among the three groups (all *P* > 0.05). Postoperative pain decreased significantly over time in all groups; however, the Bio-C Temp group consistently demonstrated the lowest VAS scores. At 24 hours, mean VAS scores were 45.5 ± 9.6, 41.1 ± 10.5, and 42.0 ± 8.9 for calcium hydroxide, Bio-C Temp, and Odontopaste, respectively (*P* = 0.030). At 48 hours, Bio-C Temp showed significantly lower pain (16.1 ± 8.4) than calcium hydroxide (26.9 ± 9.6) and Odontopaste (20.9 ± 8.9) (*P* < 0.001). Similar findings were observed at 72 hours (16.3 ± 9.8, 8.1 ± 8.1, and 9.5 ± 7.5, respectively; *P* < 0.001) and seven days (10.5 ± 8.4, 4.9 ± 6.3, and 8.8 ± 8.5, respectively; *P* < 0.001). Linear mixed-effects analysis demonstrated significant effects of the treatment group (*P* < 0.001), time (*P* < 0.001), and group × time interaction (*P* = 0.023). Bio-C Temp required significantly fewer analgesic tablets (median: 2) than calcium hydroxide (median: 3) (*P* < 0.001) and showed the longest median time to the first analgesic intake (16.4 hours). It also had the lowest incidence of interappointment flare-up, postoperative swelling, and percussion tenderness. Multivariable regression identified that Bio-C Temp (*B* = -7.789, *P* < 0.001) and Odontopaste (*B* = -6.308, *P* < 0.001) were independent predictors of lower postoperative pain than calcium hydroxide.

Conclusions: All three intracanal medicaments effectively reduced postoperative pain after nonsurgical root canal treatment. However, Bio-C Temp demonstrated superior clinical performance, providing significantly lower pain scores, reduced analgesic consumption, delayed analgesic requirements, and fewer postoperative complications than calcium hydroxide and Odontopaste. Therefore, Bio-C Temp may be considered a promising intracanal medicament for improving postoperative patient comfort during endodontic treatment.

## Introduction

Postoperative pain remains one of the most common undesirable outcomes of nonsurgical root canal treatment, affecting patient comfort, quality of life, and perception of treatment success [1]. Despite significant advances in endodontic instrumentation, irrigation protocols, and obturation techniques, postoperative pain continues to occur during the first few days after treatment, decreasing substantially within one week [2]. The etiology of post-endodontic pain is multifactorial and includes microbial persistence within the root canal system, extrusion of infected debris, mechanical irritation of periapical tissues, chemical injury caused by irrigants, and the host inflammatory response [3]. Therefore, effective control of intracanal infection between treatment visits is essential to minimize postoperative discomfort and improve clinical outcomes.

Intracanal medicaments are widely employed to eliminate residual microorganisms that survive biomechanical preparation and suppress reinfection during interappointment periods [4]. Calcium hydroxide has long been considered the gold standard because of its high alkalinity and broad antimicrobial properties [5]. However, its limited effectiveness against resistant microorganisms such as *Enterococcus faecalis *and its inability to consistently prevent postoperative pain have prompted the development of new intracanal medicaments [4]. Bio-C Temp (Angelus Indústria de Produtos Odontológicos S.A., Londrina, Brazil) is a ready-to-use bioceramic intracanal medicament with excellent biocompatibility, sustained alkaline pH, calcium ion release, and bioactive properties that may enhance periapical healing, while providing superior antimicrobial activity [6]. Odontopaste (Australian Dental Manufacturing Pty. Ltd., Brisbane, QLD, Australia), a corticosteroid-antibiotic paste containing clindamycin hydrochloride and triamcinolone acetonide, has also been advocated for reducing postoperative inflammation and pain through its combined antimicrobial and anti-inflammatory actions [7].

Although previous studies have individually evaluated these medicaments, direct comparisons of their influence on postoperative pain intensity, analgesic consumption, and time to first analgesic intake remain limited [5-7]. Therefore, this study aimed to compare the effectiveness of calcium hydroxide, Bio-C Temp, and Odontopaste as intracanal medicaments in reducing postoperative pain following nonsurgical root canal treatment. The objectives of this study were to compare postoperative pain intensity among the three intracanal medicament groups at 24 hours, 48 hours, 72 hours, and seven days using the visual analog scale (VAS); evaluate the time to first analgesic intake and the total analgesic consumption following treatment; compare the incidence of postoperative flare-ups, swelling, and percussion tenderness among the study groups; and determine the overall effectiveness of each intracanal medicament in improving postoperative patient comfort after nonsurgical root canal treatment.

## Materials and methods

Study design and setting

This prospective comparative clinical study was conducted in the Department of Conservative Dentistry and Endodontics, Yogita Dental College and Hospital, Khed, Mumbai, Maharashtra, India, between July 2025 and March 2026. Ethical approval for the study was obtained from the Institutional Ethics Committee of the Yogita Dental College and Hospital before participant recruitment (Ref No.: YDCH/IEC/2182/0Y15/2025). The study was conducted in accordance with the ethical principles of the Declaration of Helsinki (2013 revision), and written informed consent was obtained from all participants before enrollment.

The choice of intracanal medicament was made by the treating clinician as part of routine clinical practice and was independent of the investigators. No randomization, treatment allocation, or intervention by the investigators was performed. Participants were prospectively enrolled according to the medicament received during routine treatment, and the investigators were responsible only for recording postoperative pain and other clinical outcomes using a predefined study protocol.

Sample size calculation

The sample size was estimated using G*Power software version 3.1.9.7 (Heinrich-Heine-Universität Düsseldorf, Düsseldorf, Germany). Based on a previous study reporting a medium effect size (Cohen's *f* = 0.25) for postoperative pain following intracanal medicament placement, a minimum sample of 162 participants was required to achieve 80% statistical power with a two-sided significance level of 5% (*α* = 0.05) for comparison among the three groups using one-way analysis of variance (ANOVA) [8]. To compensate for an anticipated 10% attrition rate, the final sample size was increased to 180 participants (60 per group), ensuring adequate statistical power for the primary outcome of postoperative pain intensity.

Participant selection

Patients reporting to the outpatient clinics of the Department of Conservative Dentistry and Endodontics during the study period were consecutively screened for eligibility. Pulpal and periapical diagnoses were established based on clinical history, clinical examination, pulp sensibility testing, percussion and palpation tests, and radiographic findings. Irreversible pulpitis was diagnosed when clinical findings indicated an inflamed pulp incapable of predictable recovery, based on the patient’s symptoms and pulp sensibility response. Pulp necrosis was diagnosed when the tooth demonstrated no response to pulp sensibility testing, with the diagnosis correlated with clinical and radiographic findings. Periapical diagnosis was established based on percussion/palpation findings and radiographic examination. The eligibility criteria are summarized in Table 1.

**Table 1:** Inclusion and exclusion criteria for study participants.

Inclusion criteria	Exclusion criteria
Patients aged 18-60 years	Pregnant or lactating women
Patients requiring primary nonsurgical root canal treatment	Patients with systemic diseases affecting pain perception or wound healing (e.g., uncontrolled diabetes, immunocompromised conditions)
Permanent teeth diagnosed with symptomatic or asymptomatic irreversible pulpitis	Use of corticosteroids, antibiotics, analgesics, or immunosuppressive medications within the preceding week
Permanent teeth with pulp necrosis associated with apical periodontitis requiring intracanal medication	Known allergy or hypersensitivity to any study medication or material
Previously initiated endodontic treatment requiring completion with intracanal medication	Teeth with root fractures, internal resorption, or external resorption
Teeth that were restorable and had a favorable prognosis	Teeth with periodontal pockets >5 mm or advanced periodontal disease
Patients willing to provide written informed consent and comply with scheduled follow-up visits	Teeth with previously completed root canal treatment (retreatment cases)

Assignment of study groups

Radiographic examination was performed using standardized periapical radiographs to assess periapical status, root morphology, canal anatomy, and the presence or absence of periapical radiolucency. Cases requiring multivisit root canal treatment were selected when complete chemomechanical preparation and obturation could not appropriately be completed in a single appointment because of the clinical condition of the tooth, persistent exudation, symptomatic periapical inflammation, or the need for additional interappointment disinfection. Intracanal medicament was therefore placed only in cases planned for multivisit treatment and was not considered routinely indicated for all teeth with irreversible pulpitis.

This study employed a non-randomized consecutive allocation design. A total of 180 eligible participants were enrolled consecutively as they presented to the clinic and were equally assigned to three treatment groups, with 60 participants in each group, based on the intracanal medicament placed after completion of biomechanical preparation. Group I received calcium hydroxide paste, Group II received Bio-C Temp, and Group III received Odontopaste. Before initiating treatment, detailed baseline demographic and clinical information was recorded for each participant, including age, sex, tooth type (anterior, premolar, or molar), dental arch (maxillary or mandibular), pulpal diagnosis, periapical diagnosis, preoperative pain intensity measured using the VAS, and presence or absence of percussion tenderness. These baseline variables were documented to evaluate the homogeneity of the study groups and ensure valid comparisons of postoperative outcomes among the three intracanal medicament groups.

Clinical procedure

All patients underwent standardized nonsurgical root canal treatment under local anesthesia using 2% lidocaine with 1:100,000 epinephrine (Lignox® 2% A, Warren Pharmaceuticals Pvt. Ltd., Mumbai, India). After rubber dam isolation, conventional endodontic access cavities were prepared using sterile diamond burs with copious water cooling. The working length was established using an electronic apex locator (Root ZX II; J. Morita Corp., Kyoto, Japan) and confirmed radiographically using a digital radiographic system.

Biomechanical preparation was completed using the ProTaper Gold rotary file system (Dentsply Sirona Endodontics, Ballaigues, Switzerland) operated with an endodontic motor, according to the manufacturer's recommendations. The final apical preparation was completed to size F2 (25/0.08 taper) in all canals, unless anatomical considerations required modification of the final preparation size. During biomechanical preparation, the root canals were irrigated intermittently with 3% sodium hypochlorite (Prime Dental Products Pvt. Ltd., Mumbai, India) between instrumentation steps using a sterile side-vented irrigation needle. Following completion of preparation, a final rinse with 17% ethylenediaminetetraacetic acid (EDTA) (Prevest DenPro Ltd., Jammu, India) was performed for one minute to remove the smear layer, followed by a final flush with sterile saline. Root canals were dried using sterile paper points. Following canal preparation, the allocated intracanal medicament was placed into the canal using a Lentulo spiral or the manufacturer-recommended delivery system, as appropriate for the respective medicament, until complete canal filling was achieved.

Group I received calcium hydroxide paste (RC Cal, Prime Dental Products Pvt. Ltd., Mumbai, India). Group II received Bio-C Temp, a ready-to-use calcium silicate-based bioceramic intracanal medication. Group III received Odontopaste, containing clindamycin hydrochloride and triamcinolone acetonide. The access cavity was sealed using temporary restorative material (Cavit-G; 3M ESPE, Seefeld, Germany). Participants were instructed to avoid chewing on the treated tooth until the subsequent visit and were prescribed ibuprofen 400 mg every 6 hours as required as rescue medication if postoperative pain became intolerable. The number of analgesic tablets consumed and the time of first analgesic intake were recorded for each participant.

Outcome assessment

Postoperative pain intensity was evaluated using a 100-mm VAS, where 0 represented no pain and 100 represented the worst imaginable pain [9]. Baseline (preoperative) pain intensity was recorded immediately before administration of local anesthesia and commencement of endodontic treatment, after completion of the clinical and radiographic diagnostic assessment. No modifications were made to the scale, and therefore no additional permission was required. The participants independently recorded their pain scores at 24 hours, 48 hours, 72 hours, and seven days after treatment. Secondary outcome measures included the total number of analgesic tablets consumed during the follow-up period, time to first analgesic intake, occurrence of interappointment flare-ups, presence of postoperative swelling, and percussion tenderness at the recall visit. The participants were instructed to record the number and timing of rescue analgesic tablets taken throughout the follow-up period using a standardized pain diary.

At the second treatment visit, scheduled seven days after the initial treatment visit, the temporary restoration was removed, and the intracanal medicament was eliminated by copious irrigation with 3% sodium hypochlorite and 17% EDTA, accompanied by gentle circumferential instrumentation using a size 25 K-file to ensure complete removal. The root canals were then dried with sterile paper points and obturated using gutta-percha cones in combination with an AH Plus root canal sealer (Dentsply Sirona) using the cold lateral condensation technique. Following obturation, the access cavity was permanently restored with resin composite restoration according to standard clinical protocols, and the patients were scheduled for routine postoperative evaluation. 

Complete removal of the intracanal medicament was confirmed by visual inspection of the canal walls under magnification and by inspection of the final irrigation, with no visible residual medicament before obturation. This was an open-label, non-randomized prospective clinical study. Neither the treating clinician nor the participants were blinded to the intracanal medicament used. The treating clinician was aware of the intracanal medicament used because of differences in the materials and their application. However, postoperative pain data were coded, and the investigator responsible for data entry/statistical analysis was blinded to group allocation.

Statistical analysis

Each participant was assigned a unique study identification code, and personal identifiers were not included in the analytical dataset. Clinical and questionnaire data were coded numerically and entered into a password-protected electronic database accessible only to the study investigators. The dataset used for statistical analysis contained study identification codes rather than names or other directly identifying information. All records were handled confidentially and were used solely for research purposes.

Statistical analysis was performed using IBM SPSS Statistics, version 25.0 (IBM Corp., Armonk, NY). Data normality was assessed using the Shapiro-Wilk test. Continuous variables were expressed as mean ± standard deviation or median (interquartile range), as appropriate. Intergroup comparisons were performed using one-way ANOVA or the Kruskal-Wallis test with Dunn-Bonferroni post hoc analysis. Categorical variables were analyzed using the chi-square test or Fisher's exact test. Changes in postoperative pain over time were evaluated using a linear mixed-effects model with participant as a random effect and treatment group and time as fixed effects. Kaplan-Meier survival analysis with the log-rank test was used to compare the time to first analgesic intake.

Multivariable linear regression analysis was performed to identify independent predictors of postoperative pain after adjustment for potential confounding variables, including age, sex, tooth type, preoperative pain, intracanal medicament, pulpal diagnosis, and periapical diagnosis. For regression analysis, pulpal diagnosis was coded as symptomatic irreversible pulpitis (1) versus all other pulpal diagnoses (0), whereas periapical diagnosis was coded as symptomatic apical periodontitis (1) versus all other periapical diagnoses (0). Intracanal medicament and tooth type were entered as dummy variables using calcium hydroxide and anterior teeth as the respective reference categories. A two-tailed *P* < 0.05 was considered statistically significant. 

## Results

A total of 180 participants completed the study and were included in the final analysis, with 60 participants each in the calcium hydroxide, Bio-C Temp, and Odontopaste groups. Baseline demographic and clinical characteristics were comparable among the three study groups (Table 2).

**Table 2:** Baseline demographic and clinical characteristics of the study participants according to intracanal medicament groups. Data are presented as mean ± standard deviation, median (interquartile range), or frequency (percentage). One-way analysis of variance (*F*-value) was used for continuous variables, the Kruskal-Wallis test (*H*-value) for non-normally distributed continuous variables, and the chi-square test (*χ*² value) for categorical variables; *P* < 0.05 was considered statistically significant SD, standard deviation; VAS, visual analog scale; IQR, interquartile range

Variable		Calcium hydroxide (*n* = 60)	Bio-C Temp (*n* = 60)	Odontopaste (*n* = 60)	Test value	*P*-value
Age (mean ± SD)	Years	36.3 ± 9.9	38.2 ± 9.9	39.0 ± 10.9	*F* = 1.02	0.361
Sex, *n* (%)	Male	24 (40.0)	28 (46.7)	27 (45.0)	*χ*² = 0.587	0.746
	Female	36 (60.0)	32 (53.3)	33 (55.0)		
Tooth type, *n* (%)	Anterior	17 (28.3)	9 (15.0)	14 (23.3)	*χ*² = 4.72	0.317
	Premolar	16 (26.7)	16 (26.7)	20 (33.3)		
	Molar	27 (45.0)	35 (58.3)	26 (43.3)		
Arch, *n* (%)	Maxillary	38 (63.3)	37 (61.7)	28 (46.7)	*χ*² = 4.13	0.127
	Mandibular	22 (36.7)	23 (38.3)	32 (53.3)		
Pulpal diagnosis, *n* (%)	Symptomatic irreversible pulpitis	25 (41.7)	24 (40.0)	24 (40.0)	*χ*² = 2.16	0.904
	Asymptomatic irreversible pulpitis	14 (23.3)	10 (16.7)	12 (20.0)		
	Pulp necrosis with apical periodontitis	16 (26.7)	18 (30.0)	15 (25.0)		
	Previously untreated (retreat medicament)	5 (8.3)	8 (13.3)	9 (15.0)		
Periapical diagnosis, *n* (%)	Normal periapex	13 (21.7)	19 (31.7)	21 (35.0)	*χ*² = 11.27	0.08
	Symptomatic apical periodontitis	10 (16.7)	12 (20.0)	16 (26.7)		
	Asymptomatic apical periodontitis	33 (55.0)	21 (35.0)	21 (35.0)		
	Chronic apical abscess	4 (6.7)	8 (13.3)	2 (3.3)		
Preoperative pain, median (IQR)	VAS	38.5 (29.8-57.8)	36.5 (23.8-57.3)	37.0 (26.0-44.3)	*H* =1.99	0.369
Percussion tenderness, *n* (%)	Present	20 (33.3)	27 (45.0)	21 (35.0)	*χ*² = 2.03	0.362

No participant was lost to follow-up, and complete clinical and postoperative pain data were available for all study participants. The baseline demographic and clinical characteristics were comparable among the three groups. There were no statistically significant differences in age (*P* = 0.361), sex distribution (*P* = 0.746), tooth type (*P* = 0.317), dental arch (*P* = 0.127), pulpal diagnosis (*P* = 0.904), periapical diagnosis (*P* = 0.080), preoperative pain intensity (*P* = 0.369), or percussion tenderness (*P* = 0.362), confirming adequate baseline homogeneity and permitting a valid comparison of postoperative outcomes (Table 2).

Significant intergroup differences were observed at all the follow-up intervals. At 24 hours, the Bio-C Temp group exhibited the lowest mean VAS score (41.1 ± 10.5), followed by the Odontopaste (42.0 ± 8.9) and calcium hydroxide groups (45.5 ± 9.6) (*P* = 0.030). The differences became more pronounced at 48 hours, with Bio-C Temp recording significantly lower pain scores (16.1 ± 8.4) than calcium hydroxide (26.9 ± 9.6) and Odontopaste (20.9 ± 8.9) (*P* < 0.001). Similar findings were observed at 72 hours and seven days, with Bio-C Temp consistently demonstrating the lowest postoperative pain scores among the three groups (both *P* < 0.001) (Table 3).

**Table 3:** Comparison of postoperative VAS pain scores among the three intracanal medicament groups at different follow-up intervals. Data are presented as mean ± standard deviation; one-way analysis of variance (*F*-value) was used for intergroup comparisons at each follow-up interval. *P < 0.05 was considered statistically significant. VAS, visual analog scale; SD, standard deviation; df, degrees of freedom

Follow-up interval	Calcium hydroxide (Mean ± SD)	Bio-C Temp (Mean ± SD)	Odontopaste (Mean ± SD)	*F*-value (df = 2)	*P*-value
24 hours	45.5 ± 9.6	41.1 ± 10.5	42.0 ± 8.9	7.02	0.030*
48 hours	26.9 ± 9.6	16.1 ± 8.4	20.9 ± 8.9	37.02	<0.001*
72 hours	16.3 ± 9.8	8.1 ± 8.1	9.5 ± 7.5	23.72	<0.001*
7 days	10.5 ± 8.4	4.9 ± 6.3	8.8 ± 8.5	15.51	<0.001*

Longitudinal analysis using the linear mixed-effects model confirmed the significant effects of treatment group, time, and the interaction between group and time (Table 4).

**Table 4:** Linear mixed-effects model evaluating the effects of treatment group and time on postoperative pain scores. A linear mixed-effects model with participant as a random effect and treatment group and follow-up time as fixed effects was used. *P < 0.05 was considered statistically significant. df, degrees of freedom; η², partial eta squared

Source	df (numerator, denominator)	Mean square	*F*-value	*P*-value	Partial η²
Medicament group (between-subjects)	2, 177	3209.9	31.83	<0.001*	0.265
Time (within-subjects)	3, 531	44320.1	644.41	<0.001*	0.785
Group × Time interaction	6, 531	170.2	2.47	0.023*	0.027

The between-group effect was highly significant (*F* = 31.83, *P* < 0.001; partial *η*² = 0.265), indicating that intracanal medication significantly influenced postoperative pain. Time also demonstrated a significant effect (*F *= 644.41, *P* < 0.001; partial *η*² = 0.785), reflecting progressive pain reduction during the follow-up. Furthermore, a significant group × time interaction (*F* = 2.47, *P* = 0.023; partial *η*² = 0.027) indicated that the rate of pain reduction differed significantly among the three medicaments (Table 4).

At 24 hours, the pairwise differences between the study groups were not statistically significant after adjustment. However, from 48 hours onward, Bio-C Temp demonstrated significantly lower pain scores than calcium hydroxide and Odontopaste. At 48 hours, all pairwise comparisons were statistically significant. At 72 hours and seven days, Bio-C Temp remained significantly superior to calcium hydroxide, whereas the differences between calcium hydroxide and Odontopaste were no longer statistically significant at the later follow-up intervals (Table 5).

**Table 5:** Bonferroni-adjusted pairwise comparisons of postoperative pain scores between intracanal medicament groups. Bonferroni post hoc test (*t*-value) was used following a one-way analysis of variance. *P < 0.05 was considered statistically significant. CI, confidence interval; SE, standard error

Time	Pairwise comparison	Mean difference	SE	95% CI	*t* value	*P*-value
24 hours	Calcium hydroxide vs. Bio-C Temp	4.40	1.83	0.81-7.99	2.40	0.054
	Calcium hydroxide vs. Odontopaste	3.48	1.69	0.17-6.79	2.06	0.124
	Bio-C Temp vs. Odontopaste	-0.92	1.77	-4.39 to 2.56	-0.51	1.000
48 hours	Calcium hydroxide vs. Bio-C Temp	10.80	1.64	7.58-14.01	6.58	<0.001*
	Calcium hydroxide vs. Odontopaste	5.94	1.68	2.64-9.24	3.53	0.002*
	Bio-C Temp vs. Odontopaste	-4.85	1.57	-7.94 to -1.77	-3.08	0.008*
72 hours	Calcium hydroxide vs. Bio-C Temp	8.18	1.65	4.95-11.41	4.96	<0.001*
	Calcium hydroxide vs. Odontopaste	6.77	1.59	3.65-9.90	4.24	<0.001*
	Bio-C Temp vs. Odontopaste	-1.41	1.43	-4.21 to 1.39	-0.98	0.974
7 days	Calcium hydroxide vs. Bio-C Temp	5.61	1.35	2.95-8.26	4.14	<0.001*
	Calcium hydroxide vs. Odontopaste	1.73	1.54	-1.28 to 4.75	1.12	0.787
	Bio-C Temp vs. Odontopaste	-3.87	1.37	-6.55 to -1.19	-2.83	0.016*

Comparison of secondary postoperative outcomes among the three intracanal medicament groups was done and statistically significant differences were observed in the secondary clinical outcomes (Table 6).

**Table 6:** Comparison of secondary postoperative outcomes among the three intracanal medicament groups. Continuous data are presented as median (interquartile range), and categorical data as frequency (percentage); the Kruskal-Wallis test was used for non-normally distributed continuous variables, and the chi-square test or Fisher's exact test was used for categorical variables. *P < 0.05 was considered statistically significant. IQR, interquartile range

Variable	Calcium hydroxide	Bio-C Temp	Odontopaste	Test value	*P*-value
Analgesic tablets consumed, median (IQR)	3 (2-5)	2 (1-3)	2 (1-3)	*H* = 27.22	<0.001*
Time to first analgesic intake, *h*, median (IQR)	6.1 (3.8-12.9)	16.4 (6.3-34.9)	13.7 (6.6-22.8)	*H* = 19.51	<0.001*
Interappointment flare-up, *n* (%)	8 (13.3)	1 (1.7)	2 (3.3)	*χ*² = 8.32	0.016*
Postoperative swelling, *n* (%)	10 (16.7)	2 (3.3)	4 (6.7)	*χ*² = 7.13	0.028*
Percussion tenderness at recall, *n* (%)	16 (26.7)	5 (8.3)	14 (23.3)	*χ*² = 7.30	0.026*

The Bio-C Temp group required significantly fewer analgesic tablets than the calcium hydroxide group (median: 2 vs. 3 tablets; *P* < 0.001) and demonstrated the longest median time to first analgesic intake (16.4 hours), compared with Odontopaste (13.7 hours) and calcium hydroxide (6.1 hours) (*P* < 0.001). Interappointment flare-ups occurred most frequently in the calcium hydroxide group, whereas Bio-C Temp had the lowest incidence (*P* = 0.016). Similarly, postoperative swelling (*P* = 0.028) and percussion tenderness at recall (*P* = 0.026) were significantly less frequent in the Bio-C Temp group than in the other two treatment groups (Table 6).

Table 7 shows that Bio-C Temp demonstrated significantly lower postoperative pain scores than calcium hydroxide at all follow-up intervals (adjusted *P* < 0.05). Odontopaste also showed significantly lower pain scores than did calcium hydroxide at most follow-up intervals, whereas no significant difference was observed between the Bio-C Temp and Odontopaste groups.

**Table 7:** Dunn's pairwise comparisons of postoperative pain scores among the three intracanal medicament groups. Dunn's post hoc test with Bonferroni adjustment (*z*-value) was performed following the Kruskal-Wallis test. *P < 0.05 was considered statistically significant.

Dunn’s pairwise comparison	Tablets consumed (Bonferroni)		Time to first analgesic (Bonferroni)	
	Test value (*z*)	*P*-value	Test value (*z*)	*P*-value
Calcium hydroxide vs. Bio-C Temp	5.07	<0.001*	-4.28	<0.001*
Calcium hydroxide vs. Odontopaste	3.59	0.001*	-3.06	0.007*
Bio-C Temp vs. Odontopaste	-1.47	0.420	1.22	0.666

**Figure 1:**
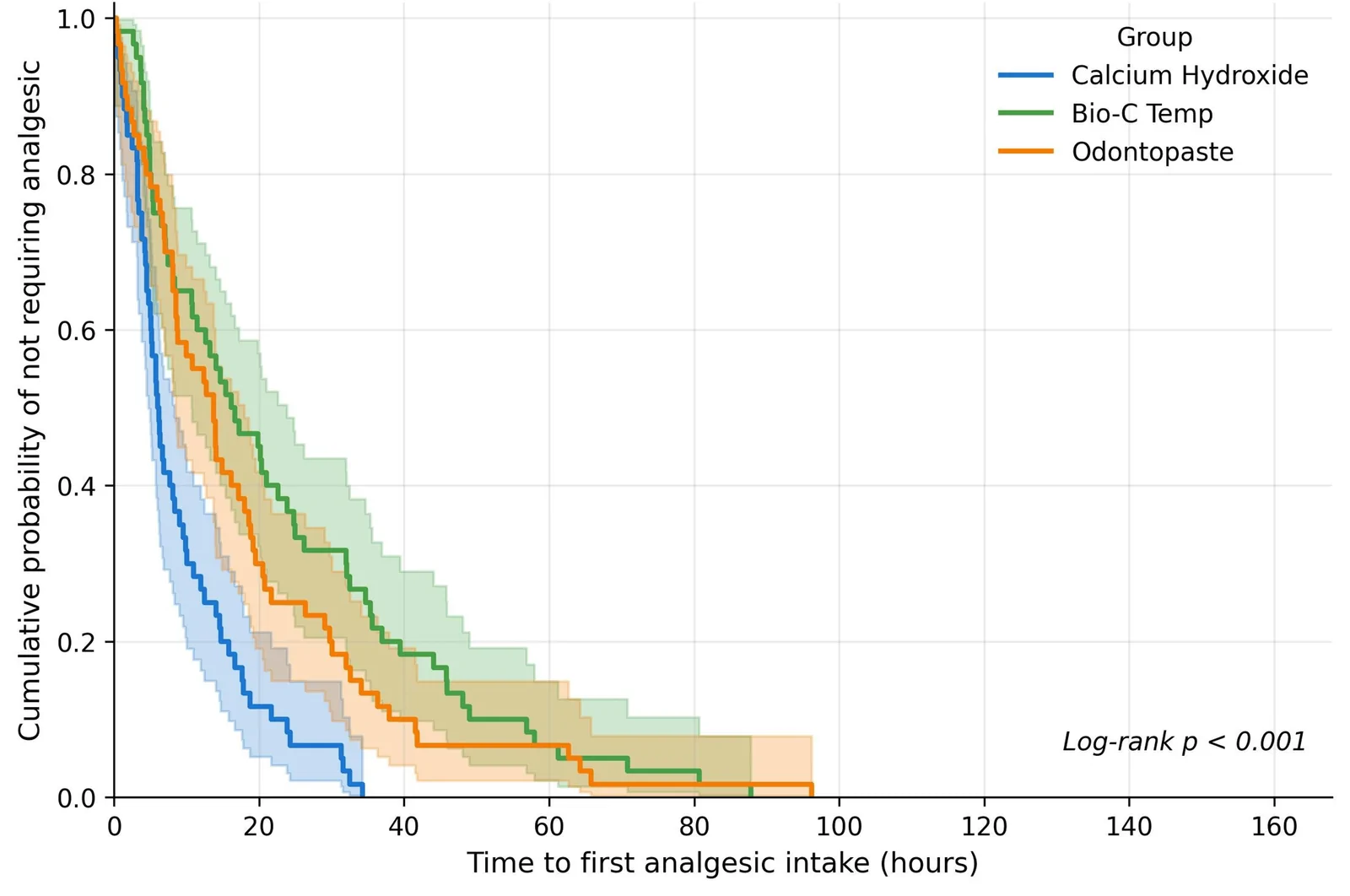
Kaplan-Meier curve for time to first analgesic intake by intracanal medicament group. Curves were generated by the authors using IBM SPSS Statistics, version 25.0 (IBM Corp., Armonk, NY) from the study data. Shaded areas represent 95% confidence intervals. Differences between groups were compared using the log-rank test (*P* < 0.001).

Kaplan-Meier survival analysis (Figure 1) demonstrated significant differences in the time to first analgesic intake among the three intracanal medicaments (log-rank *P* < 0.001). Participants treated with Bio-C Temp exhibited the longest analgesic-free interval throughout the postoperative period, whereas those treated with calcium hydroxide required rescue analgesics considerably earlier. The Odontopaste group showed an intermediate survival rate. These findings further support the superior postoperative pain control achieved with the Bio-C Temp.

Multivariable linear regression analysis identified intracanal medication as the principal independent predictor of postoperative pain at 72 hours (Table 8).

**Table 8:** Multivariable linear regression analysis identifying predictors of postoperative pain at 72 hours. Multivariable linear regression analysis was performed. *P < 0.05 was considered statistically significant. B, unstandardized regression coefficient; CI, confidence interval; SE, standard error; VAS, visual analog scale

Predictor variable	B	SE	95% CI	*P*-value
Constant	19.785	3.595	12.687-26.882	<0.001*
Age (years)	-0.107	0.064	-0.233 to 0.020	0.098
Sex (male vs. female)	0.546	1.319	-2.058 to 3.149	0.680
Tooth type: molar (vs. anterior)	-1.103	1.687	-4.433 to 2.228	0.514
Tooth type: premolar (vs. anterior)	-1.205	1.845	-4.847 to 2.437	0.515
Symptomatic irreversible pulpitis (vs. other)	0.996	1.336	-1.641 to 3.633	0.457
Symptomatic apical periodontitis (vs. other)	-0.194	1.597	-3.347 to 2.959	0.904
Preoperative pain (VAS)	0.019	0.033	-0.046 to 0.085	0.560
Bio-C Temp (vs. calcium hydroxide)	-7.789	1.600	-10.947 to -4.631	<0.001*
Odontopaste (vs. calcium hydroxide)	-6.308	1.609	-9.485 to -3.131	<0.001*

After adjustment for age, sex, tooth type, pulpal diagnosis, periapical diagnosis, and preoperative pain, treatment with Bio-C Temp (*B* = -7.789, *P* < 0.001) and Odontopaste (*B* = -6.308, *P* < 0.001) remained significantly associated with lower postoperative pain than treatment with calcium hydroxide. None of the remaining demographic or clinical variables showed a statistically significant association with postoperative pain (all *P* > 0.05), as shown in Table 8.

## Discussion

This prospective comparative clinical study evaluated the effectiveness of three intracanal medicaments, calcium hydroxide, Bio-C Temp, and Odontopaste, in controlling postoperative pain following nonsurgical root canal treatment. The findings demonstrated that, although pain decreased progressively over time in all treatment groups, Bio-C Temp consistently provided superior postoperative pain control, reduced analgesic consumption, delayed the need for rescue analgesics, and resulted in fewer postoperative complications than calcium hydroxide and Odontopaste. These observations indicate that the selection of intracanal medicaments plays a significant role in improving patient comfort during the interappointment period [4].

The baseline demographic and clinical variables were comparable among the three groups, confirming that postoperative differences were attributable to intracanal medicaments rather than confounding patient- or tooth-related factors. The most important finding of the present investigation was the significantly lower postoperative pain scores observed in the Bio-C Temp group at days 24, 48, 72, and 7. Postoperative pain after root canal treatment primarily results from persistent intracanal microorganisms, extrusion of infected debris beyond the apex, and an inflammatory response of periapical tissues [3].

Bio-C Temp is a premixed, calcium silicate-based bioceramic intracanal medicament that maintains a prolonged alkaline pH through continuous calcium hydroxide release while exhibiting excellent sealing ability, calcium ion release, and biocompatibility [10]. These properties enhance the antimicrobial activity and reduce periapical inflammation, thereby contributing to superior pain reduction. The sustained release of hydroxyl ions creates an unfavorable environment for bacterial survival, including resistant species such as *Enterococcus faecalis*, while simultaneously promoting mineralization and tissue healing [11].

Similar findings have been reported in previous studies [6,11]. A recent in vitro study by Balto et al. [12] demonstrated that Bio-C Temp exhibited significantly greater antibiofilm efficacy against *Fusobacterium nucleatum* than calcium hydroxide after two weeks of application, with 75.25% bacterial death compared with 58.65% for calcium hydroxide. Although the triple antibiotic paste achieved the highest antibacterial effect, Bio-C Temp significantly outperformed calcium hydroxide, supporting its superior antimicrobial potential. This enhanced disinfection may have contributed to the lower postoperative pain scores, reduced analgesic consumption, and fewer flare-ups observed in this study [12]. 

Odontopaste also demonstrated significantly better pain reduction than calcium hydroxide, although its effectiveness was inferior to that of Bio-C Temp during the later follow-up periods. The favorable performance of Odontopaste may be attributed to its combination of clindamycin hydrochloride and triamcinolone acetonide [7,13]. Clindamycin effectively suppresses residual intracanal bacteria, whereas triamcinolone reduces the inflammatory response by inhibiting prostaglandin synthesis and decreasing vascular permeability [13]. Abbott [14] previously reported that corticosteroid-antibiotic intracanal medicaments provide rapid postoperative pain relief, particularly during the early postoperative period. The present results support these observations, although the advantage diminished over time as the anti-inflammatory effects subsided.

Plutzer et al. [15] demonstrated that calcium hydroxide reduced the viability of *E. faecalis* biofilms by more than 99%, whereas Odontopaste alone showed no significant antibiofilm effect. Interestingly, combining Odontopaste with calcium hydroxide markedly enhanced bacterial killing, suggesting that calcium hydroxide remained the principal antimicrobial component [15]. These findings support the continued use of calcium hydroxide as an effective intracanal medicament, although the superior clinical outcomes achieved with Bio-C Temp in the present study may be related to its calcium silicate-based composition and prolonged bioactive ion release, rather than antimicrobial activity alone.

Calcium hydroxide demonstrated the highest postoperative pain scores throughout the study despite being regarded as the conventional gold standard intracanal medicament. Although its high pH provides broad-spectrum antimicrobial activity, calcium hydroxide exhibits limited effectiveness against resistant microorganisms such as *E. faecalis* and *Candida albicans* [16]. Furthermore, the buffering capacity of dentin may reduce hydroxyl ion diffusion, thereby limiting its antibacterial action within complex root canal anatomy. These limitations may explain the comparatively higher incidence of postoperative pain observed in the present study. Similar findings have been reported in clinical studies, in which calcium hydroxide was associated with greater postoperative discomfort than newer intracanal medicaments [17,18]. Ahmad et al. [18] reported that while calcium hydroxide significantly reduced postoperative pain compared to no dressing, several alternative medicaments demonstrated superior analgesic effects.

Another clinically important observation was the significantly lower analgesic consumption and longer time to first analgesic intake among the patients treated with Bio-C Temp. Reduced analgesic requirements directly reflect improved postoperative comfort and indicate a more effective control of inflammation during the healing period. Likewise, the lower incidence of interappointment flare-ups, postoperative swelling, and percussion tenderness in the Bio-C Temp group further supports its superior biological behavior and enhanced control of residual infections. These findings are consistent with previous reports suggesting that bioceramic materials minimize periapical irritation owing to their excellent biocompatibility and bioactive properties [19].

The longitudinal analysis further demonstrated significant effects of both treatment group and time, together with a significant group × time interaction, indicating that postoperative pain reduction differed according to the intracanal medication used. Multivariable regression analysis confirmed that the choice of intracanal medication was the only significant independent predictor of postoperative pain after adjustment for demographic and clinical variables. This finding emphasizes that material selection may have a greater influence on postoperative pain than patient age, sex, tooth type, or pre-operative diagnosis.

Nevertheless, some studies have reported no significant differences in postoperative pain among various intracanal medicaments with respect to postoperative pain [18,20]. These discrepancies may be explained by differences in the study design, instrumentation systems, irrigation protocols, operator experience, sample size, pain assessment methods, duration of follow-up, and variations in pulpal and periapical diagnoses. In addition, several earlier investigations evaluated only calcium hydroxide and corticosteroid-containing medicaments, whereas evidence regarding newer premixed bioceramic intracanal medicaments remains relatively limited [12,18].

Clinical implications

The findings of this study suggest that the selection of intracanal medicaments can substantially influence postoperative patient comfort following nonsurgical root canal treatment. Bio-C Temp demonstrated superior pain control, reduced analgesic consumption, fewer postoperative complications, and prolonged analgesic-free intervals than calcium hydroxide and Odontopaste. These characteristics make Bio-C Temp a promising intracanal medicament, particularly in patients presenting with symptomatic teeth or those at greater risk of postoperative pain. Incorporating bioceramic intracanal medicaments into routine endodontic practice may improve patient satisfaction and treatment acceptance while enhancing the quality of postoperative care.

Limitations

This study has several limitations. As a nonrandomized, single-center observational investigation, the findings may be subject to selection bias and residual confounding, and they may not be fully generalizable to other populations or clinical settings. The absence of randomization and allocation concealment could have introduced systematic differences between groups despite comparable baseline characteristics. The follow-up period was limited to seven days, precluding assessment of long-term periapical healing and treatment success. Postoperative pain was evaluated using patient-reported VAS scores, which remain subjective despite being widely accepted. Furthermore, microbiological evaluation of bacterial reduction and radiographic assessment of periapical healing were not performed. Future multicenter randomized clinical trials with larger sample sizes, longer follow-up periods, microbiological analyses, and radiographic outcome assessments are warranted to further validate the long-term clinical benefits of Bio-C Temp and other contemporary intracanal medicaments.

## Conclusions

Within the limitations of this prospective, nonrandomized clinical study, Bio-C Temp was associated with lower postoperative pain following nonsurgical root canal treatment compared with calcium hydroxide and Odontopaste. Patients receiving Bio-C Temp showed lower pain intensity, reduced analgesic consumption, delayed analgesic requirements, and fewer postoperative complications during follow-up. Although all three intracanal medicaments were associated with progressive pain reduction, Bio-C Temp showed the most favorable observed clinical outcomes. These findings suggest a potential benefit of Bio-C Temp for postoperative patient comfort, which should be confirmed in well-designed randomized controlled studies.
